# Error-based assessment of technical performance in pediatric laparoscopic inguinal hernia repair using the generic error rating tool: a retrospective observational study

**DOI:** 10.1007/s00464-025-12022-7

**Published:** 2025-07-18

**Authors:** Yohei Sanmoto, Akio Kawami, Masanaga Matsumoto, Kouji Masumoto

**Affiliations:** 1 Department of Pediatric Surgery, University of Tsukuba Hospital, 2-1-1 Amakubo, Tsukuba, 305-8576 Japan; 2 Graduate School of Comprehensive Human Sciences, University of Tsukuba, Tsukuba, Japan

**Keywords:** Error analysis, Inguinal hernia, Pediatrics, Laparoscopy, Skill assessment, Surgical education

## Abstract

**Background:**

We aimed to evaluate the feasibility, reliability, and educational utility of applying the generic error rating tool (GERT) for identifying intraoperative technical errors and assessing technical proficiency during pediatric single-incision laparoscopic percutaneous extraperitoneal closure (SILPEC) procedures.

**Methods:**

This retrospective observational study conducted at a single tertiary-care university hospital included 25 pediatric patients (< 16 years) who underwent unilateral SILPEC procedures with complete operative video recordings between January and December 2022. Surgical technical performance was independently assessed using the GERT and objective structured assessment of technical skill (OSATS) frameworks by two blinded pediatric surgeons. Inter-rater reliability was analyzed using intraclass correlation coefficients (ICCs). Based on the median OSATS score as a cut-off, cases were further categorized into high- and low-performance groups, and the association between GERT-derived error metrics and technical proficiency was evaluated.

**Results:**

A mean total of 273 errors and 32 events were identified across all procedures. GERT demonstrated excellent inter-rater reliability for total errors (ICC = 0.92) and total events (ICC = 0.97). GERT error counts showed a strong negative correlation with OSATS scores for both evaluators: Spearman’s ρ = − 0.78 (*P* < 0.001) for evaluator 1 and ρ = − 0.63 (*P* < 0.001) for evaluator 2. Compared with the high-performance group (OSATS > 26), the low-performance group exhibited significantly more errors (median 13 vs. 8.5) and events (median 1 vs. 0.5) per procedure (*P* = 0.0016 and *P* = 0.032, respectively). Errors and events were disproportionately concentrated during suturing along the outer semicircle of the hernia orifice.

**Conclusions:**

GERT demonstrated high feasibility and excellent inter-rater reliability for assessing technical performance in pediatric SILPEC. Its structured error profiling correlated with global performance scores and enabled detailed identification of intraoperative errors, supporting tailored formative feedback and continuous skill development in pediatric surgical education.

Laparoscopic percutaneous extraperitoneal closure (LPEC) for pediatric inguinal hernia, first introduced by Takehara et al. [1], has gained widespread acceptance owing to its minimally invasive nature, favorable cosmetic outcomes, and ability to repair contralateral patent processus vaginalis (CPPV) prophylactically, compared with the conventional open approach [2]. Subsequently, Uchida et al. [3] developed single-incision LPEC (SILPEC), which is now commonly used for pediatric inguinal hernia repair. As SILPEC is often performed by young surgeons early in their training, it presents a valuable educational opportunity, highlighting the need for a robust and objective method to assess surgical skills and provide meaningful formative feedback. In this context, our previous study demonstrated that the number of forceps grasps of the peritoneum decreases with accumulated surgical experience, suggesting its potential as an objective indicator of technical proficiency [4].

Traditional surgical education has primarily focused on the correct execution of procedures. However, increasing attention is now given to intraoperative errors as opportunities for learning [5]. Appropriate evaluation and feedback on such errors can promote higher-order cognitive skills and enhance overall performance [6, 7]. Technical errors often indicate specific skill deficiencies, and analyzing them can complement global skill assessments, helping target educational interventions. To incorporate this approach effectively into surgical training, it is crucial to systematically identify, classify, and analyze errors, underscoring the need for structured tools that can capture the nature and underlying mechanisms of these errors in detail. The generic error rating tool (GERT) is a recently developed framework designed to systematically identify and categorize intraoperative technical errors and events in laparoscopic surgery based on task characteristics and underlying mechanisms [8]. In adult laparoscopic surgery, GERT has enabled detailed analysis of error types and mechanisms, providing a level of granularity not achievable with conventional global rating tools. This feature may facilitate more meaningful formative feedback and offer an objective reflection of technical proficiency through the frequency and nature of intraoperative errors [8–10]. Despite its promising utility, the application of GERT in pediatric laparoscopic surgery remains limited, and its feasibility and educational value in this setting have not been fully explored.

Therefore, this study aimed to evaluate the feasibility and effectiveness of assessing intraoperative performance during pediatric SILPEC procedures by extracting and analyzing technical errors and events using GERT. Demonstrating its effectiveness in this context could establish GERT as an objective framework for performance assessment, contributing to the advancement of surgical education in pediatric surgery.

## Materials and methods

### Study design and patients

This retrospective observational study included male patients aged < 16 years who underwent SILPEC at the Department of Pediatric Surgery at our hospital, Japan, between January 2022 and December 2022. Only cases with complete surgical video recordings were included in this study. To ensure consistent evaluation of the entire surgical process, only unilateral procedures were considered, and bilateral procedures, including those for asymptomatic CPPV, were excluded to maintain comparability. Patient characteristics, such as age, height, weight, body mass index, laterality of surgery, presence of associated hydrocele, and history of hernia incarceration, were reviewed. Surgical outcomes, including procedure time and hernia recurrence within 1 year postoperatively, were retrospectively reviewed. Procedure time was defined as the duration from the initiation of pneumoperitoneum to the removal of forceps after completing the operation. Detailed surgical techniques for SILPEC are described in our previous report [4].

### Skill validation

Technical performance was assessed using the objective structured assessment of technical skills (OSATS), a validated global rating scale covering the following seven domains: respect for tissue, time and motion, instrument handling, knowledge of instruments, flow of operation, use of assistants, and knowledge of the specific procedure [11]. Each domain was rated on a five-point Likert scale, with higher scores reflecting better performance. Two pediatric surgeons, Y.S. and A.K., both board-certified by the Japanese Society of Pediatric Surgeons and having experience in performing over 100 SILPEC (LPEC) procedures, evaluated all assessments conducted in this study. Neither of the evaluators participated in any surgical procedures involving the patients included in this study. All video assessments were performed under complete anonymization, ensuring that evaluators had no access to identifiable information about operating surgeons. In the context of SILPEC, the assistant primarily functions as the scopist. Therefore, the “Use of Assistants” domain was interpreted as assessing the surgeon’s ability to effectively instruct the scopist and maintain an optimal visual field throughout the procedure. Each case was independently reviewed using the OSATS framework, and the average of the two scores was used as the OSATS score for that procedure. Cases were categorized into high- and low-performance groups based on the median OSATS score.

### Technical error and event analysis

GERT is a structured framework designed to evaluate technical performance in laparoscopic surgery by classifying errors into four mechanisms: excessive use of force or distance, insufficient use of force or distance, inadequate visualization, and wrong orientation of instrument [8]. Errors are further categorized into nine generic surgical tasks: abdominal access/removal of instruments or trocars, use of retractors, use of energy devices, grasping and dissection, cutting/transection/stapling, clipping, suturing, use of suction, and others. In this study, focusing on SILPEC procedures, only four task categories were applicable: abdominal access/removal of instruments or trocars, grasping, suturing, and others. For comparative analysis, a technical error was defined as “the failure of planned actions to achieve their desired goal” [12] and an event as “an action that may require additional measures to prevent an adverse outcome” [13]. Based on these definitions, technical errors included unintended instrument interference with adjacent organs, the needle, or the camera scope; slippage of graspers holding the peritoneum; and accidental withdrawal of the needle or suture. Events included bleeding, grasping of the vas deferens, inadvertent needle puncture of the peritoneum, inadvertent ‘skip’ maneuvers of the needle through the peritoneum, and peritoneal injury. The surgical videos were independently reviewed by the same two evaluators using the GERT framework. The SILPEC procedure was divided into six surgical steps (Table 1), and technical errors and events in each step were identified. For each case, the number of technical errors and events was independently assessed by both evaluators, and the mean count was used as the GERT-based score for that procedure. Notably, to minimize potential anchoring bias during formal assessments, OSATS and GERT evaluations were conducted on separate days, ensuring that each surgical video was reviewed twice, once for each scoring framework.

**Table 1:** Overview of surgical steps of single-incision laparoscopic percutaneous extraperitoneal closure

Steps	Start (S) and endpoint (E)
Step 1: Insertion of forceps	S: Initiation of pneumoperitoneum
	E: Insertion of forceps into the abdominal cavity
Step 2: Insertion of Lapaherclosure™	E: Insertion of the Lapaherclosure™ tip into the preperitoneal space
Step 3: Suturing along the outer semicircle of the hernia orifice	E: Suturing along the outer semicircle of the hernia orifice, followed by release of the suture into the abdominal cavity
Step 4: Suturing along the inner semicircle of the hernia orifice	E: Suturing along the inner semicircle of the hernia orifice, followed by grasping and externalization of the suture
Step 5: Checking for peritoneal skip	E: Completion of peritoneal skip check
Step 6: Suture tying and forceps removal	E: Removal of the forceps

### Outcome measurements

The primary outcomes were as follows: (1) number of technical errors and events identified using the GERT framework, compared between high- and low-performance groups based on OSATS scores; (2) inter-rater reliability between the two evaluators, assessed using the intraclass correlation coefficient (ICC) for error and event counts; and (3) correlation between OSATS scores and GERT scores. Secondary outcomes included comparisons of patient characteristics and surgical outcomes between the two groups. In addition, for surgeons who had performed five or more cases during the study period, we conducted a subgroup analysis to compare procedure time, OSATS scores, and GERT scores across individual surgeons.

### Rater training

Prior to the formal assessment, rater training was conducted using five surgical videos from cases not included in this study. One of the evaluators (Y.S.) had previously been involved in a research project focusing on technical error analysis using the GERT framework in endoscopic surgery and was therefore already well-acquainted with the classification system and its practical application. During training, Y.S. and A.K. jointly reviewed the five videos and engaged in detailed discussions to calibrate their interpretations of technical errors, refine the application of GERT categories, and establish consistent inclusion and exclusion criteria. This process ensured alignment in scoring approaches and contributed to the consistency and reliability of subsequent evaluations.

### Sample size calculation

After rater training, five additional surgical videos were independently assessed by the two evaluators to estimate the expected difference in technical error counts between performance groups. Based on the median OSATS scores, cases were categorized into high-performance and low-performance groups, and the mean number of technical errors was compared. The high-performance group had a mean of 7.8 errors (SD = 1.8), while the low-performance group had a mean of 14.2 errors (SD = 5.3). A power analysis was conducted using G*Power (version 3.1.9.6; Heinrich-Heine-Universität Düsseldorf, Düsseldorf, Germany) for a two-sided nonparametric test with α = 0.05 and power (1–β) = 0.8, which indicated that at least 8 cases per group were needed.

### Statistical analyses

Continuous variables are reported as medians with interquartile ranges (IQRs), and categorical variables are presented as frequencies and percentages. Differences in patient characteristics and surgical outcomes between the high-performance and low-performance groups were analyzed using the chi-square test or Fisher’s exact test for categorical variables and the Mann–Whitney U test for continuous variables. Technical errors and events identified by GERT were compared between groups using the Mann–Whitney U test. Inter-rater reliability for technical errors and events was assessed using the ICC with a two-way random-effects model and absolute agreement. The correlation between OSATS scores and GERT error scores was evaluated using Spearman’s rank correlation coefficient. Additionally, for surgeons who had performed five or more cases, procedure time, OSATS scores, and GERT scores were compared across surgeons using the Kruskal–Wallis test. A two-sided *P*-value of < 0.05 was considered statistically significant. All statistical analyses were performed using GraphPad Prism (version 10.4.0; GraphPad Inc., La Jolla, CA, USA).

### Ethics

This retrospective observational study was conducted in accordance with the principles of the Declaration of Helsinki (2024) and was approved by the Institutional Review Board of the University of Tsukuba Hospital (approval number R06-325). At our institution, all endoscopic surgeries are routinely recorded as part of standard surgical care. During the standard preoperative consent process, patients and their guardians are informed that the procedure will be video recorded and that the recordings may be used for clinical and educational purposes. Written informed consent is obtained for this video recording and its potential use. Given the retrospective nature of this study and the use of anonymized videos collected with prior consent, the IRB waived the requirement for additional written informed consent or assent specific to this research. An opt-out system was used to allow patients and their guardians the opportunity to decline participation.

## Results

A total of 45 SILPEC procedures were performed on male patients during the study period, with 19 of these involving bilateral cases, including those involving prophylactic repair for asymptomatic CPPV. Of the 26 unilateral cases, one was excluded due to the absence of a complete surgical video, yielding a final analytical cohort of 25 cases.

**Fig. 1:**
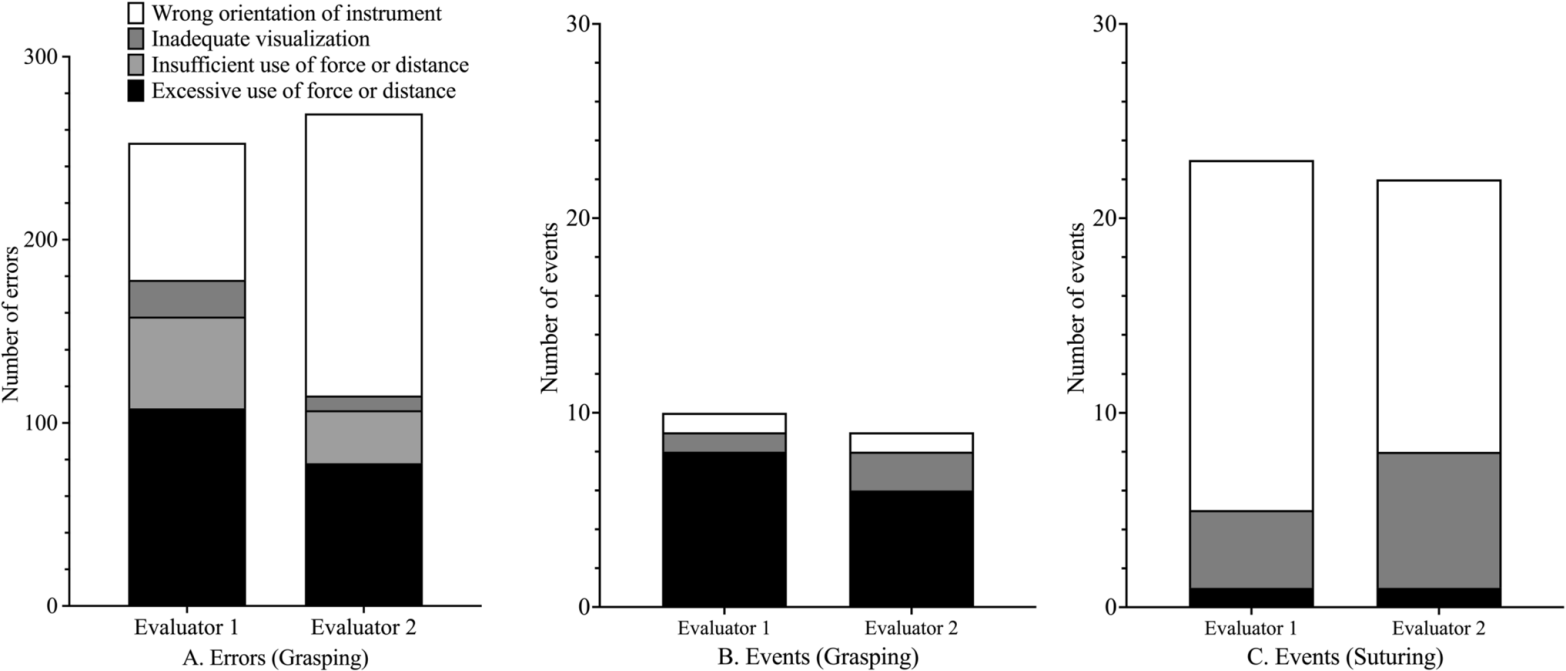
Mechanisms of intraoperative errors and events identified using the generic error rating tool. **A** Distribution of error mechanisms during grasping. **B** Distribution of event mechanisms during grasping. **C** Distribution of event mechanisms during suturing

The median (IQR) procedure time was 23.3 (20.3, 29.4) minutes, and approximately 640 min of surgical video data were analyzed. A mean total of 273 errors (267 by evaluator 1, 278 by evaluator 2) and 32 events (33 by evaluator 1, 31 by evaluator 2) were identified across all procedures (Table 2). Among the six surgical steps, Step 3 (suturing along the outer semicircle of the hernia orifice) had the highest number of errors, with a mean of 79 errors (72 by evaluator 1 [30.0%] and 86 by evaluator 2 [30.9%]). Similarly, Step 3 also had the highest number of events, with a mean of 18.5 events (19 by evaluator 1 [57.6%] and 18 by evaluator 2 [58.1%]). Inter-rater reliability was excellent for the total number of errors and total number of events, with ICCs of 0.92 and 0.97, respectively (Table 2). At the task-specific level, reliability varied: excellent agreement (ICC ≥ 0.90) was observed for grasping, moderate agreement (ICC = 0.54) for abdominal access/removal of instruments, and poor agreement for suturing (ICC = 0.11). Figure 1 illustrates the mechanisms underlying errors and events during grasping and suturing. For errors during grasping, evaluator 1 most frequently identified “excessive use of force or distance,” followed by “wrong orientation of instrument,” while evaluator 2 most frequently identified “wrong orientation of instrument,” followed by “excessive use of force or distance.” Regarding events, “excessive use of force or distance” predominated during grasping, while “wrong orientation of instrument” was the most common mechanism during suturing (Fig. 1).

**Table 2:** Interrater reliability

Task	Evaluator 1	Evaluator 2	ICC average measures
Total number of errors	267	278	0.92
Abdominal access/removal of instruments	8	2	0.54
Grasping	253	269	0.93
Suturing	6	7	0.11
Others	0	0	n.a
Total number of events	33	31	0.97
Abdominal access/removal of instruments	0	0	n.a
Grasping	10	9	0.97
Suturing	23	22	0.97
Others	0	0	n.a

*ICC* intraclass correlation coefficient, *n.a.* not applicable

Correlation analysis between GERT error scores and OSATS scores revealed a significant negative relationship for both evaluators (Spearman’s ρ = − 0.78, *P* < 0.001 for evaluator 1; ρ = − 0.63, *P* < 0.001 for evaluator 2). The median (IQR) OSATS score based on two independent evaluations was 26 (24.5, 28.5). Cases with scores ≤ 26 were classified as the low-performance group (n = 13), and scores > 26 as the high-performance group (n = 12). No significant differences in patient characteristics were found between the groups. However, the median (IQR) procedure time was significantly longer in the low-performance group [29.4 (23.5, 31.7) min] compared with the high-performance group [21.4 (19.4, 22.9) min] (*P* = 0.0045). Postoperative recurrence was observed in one patient from the low-performance group, but this difference was not statistically significant (*P* = 1) (Table 3).

**Table 3:** Comparison of patient characteristics and surgical outcomes between the high-performance and low-performance groups

	Low-performance group (OSATS ≤ 26)	High-performance group (OSATS > 26)	*P*-value
	n = 13	n = 12	
Age (months), median (IQR)	29 (15, 57)	31 (14.8, 42.3)	0.53
Height (cm), median (IQR)	87.1 (77.7, 108)	89.3 (78.6, 100.4)	0.7
Body wight (kg), median (IQR)	12.9 (10, 17)	12.5 (9.3,15.6)	0.43
BMI (kg/m^2^), median (IQR)	16 (15.5, 17.8)	15.5 (14.7, 16.7)	0.27
Left side, n (%)	3 (23.1)	5 (41.7)	1
Hydrocele, n (%)	5 (38.5)	4 (33.3)	1
History of incarceration, n (%)	2 (15.4)	0	0.48
Procedure time (min), median (IQR)	29.4 (23.5, 31.7)	21.4 (19.4, 22.9)	0.0045
Postoperative recurrence, n (%)	1 (7.7)	0	1

*IQR* interquartile range

**Fig. 2:**
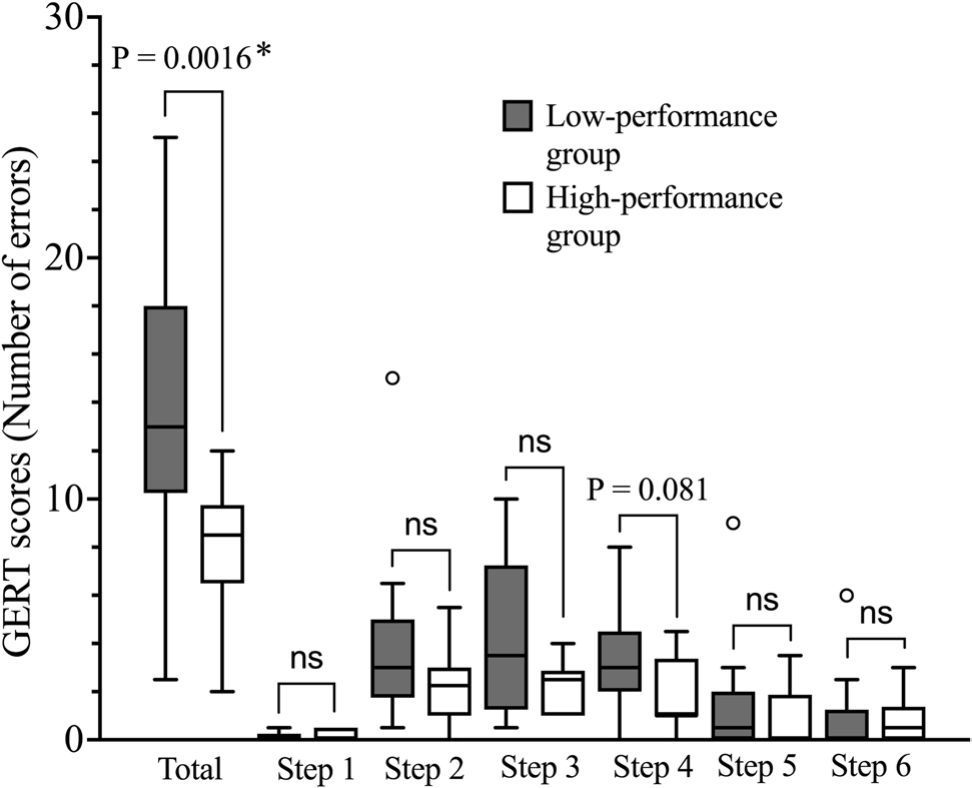
Comparison of the number of intraoperative errors between high-performance and low-performance groups based on generic error rating tool scores

**Fig. 3:**
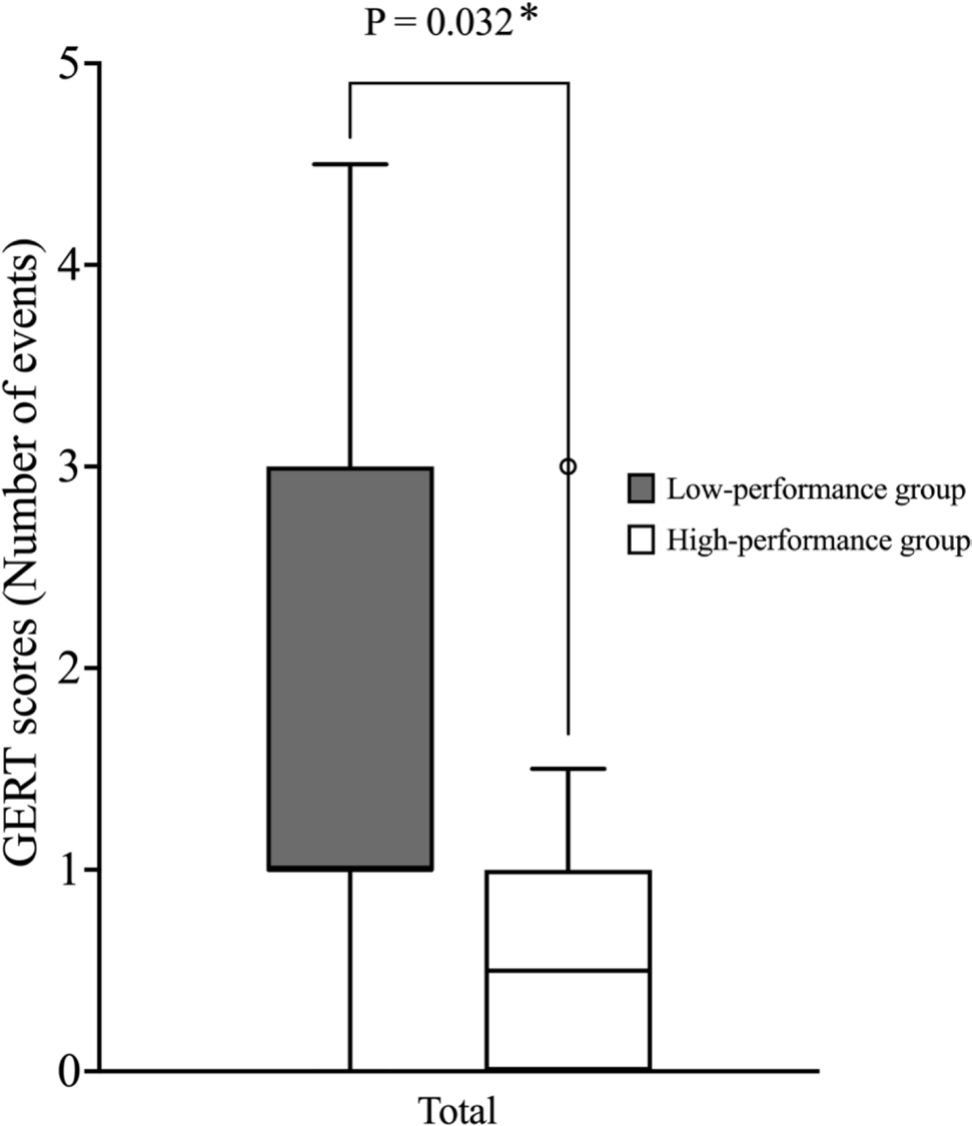
Comparison of the total number of intraoperative events between high-performance and low-performance groups based on generic error rating tool scores

The median (IQR) number of errors was significantly higher in the low-performance group [13 (11, 18)] than in the high-performance group [8.5 (6.5, 9.3)] (*P* = 0.0016) (Fig. 2). Similarly, the total number of events was also significantly greater in the low-performance group [1 (1, 2)] than in the high-performance group [0.5 (0, 1)] (*P* = 0.032) (Fig. 3). A stepwise error analysis was conducted to assess whether specific surgical steps were more error-prone based on performance level. No significant differences were observed across most steps; however, Step 4 (suturing along the inner semicircle of the hernia orifice) showed a tendency for more errors in the low-performance group (*P* = 0.081) (Fig. 2).

**Fig. 4:**
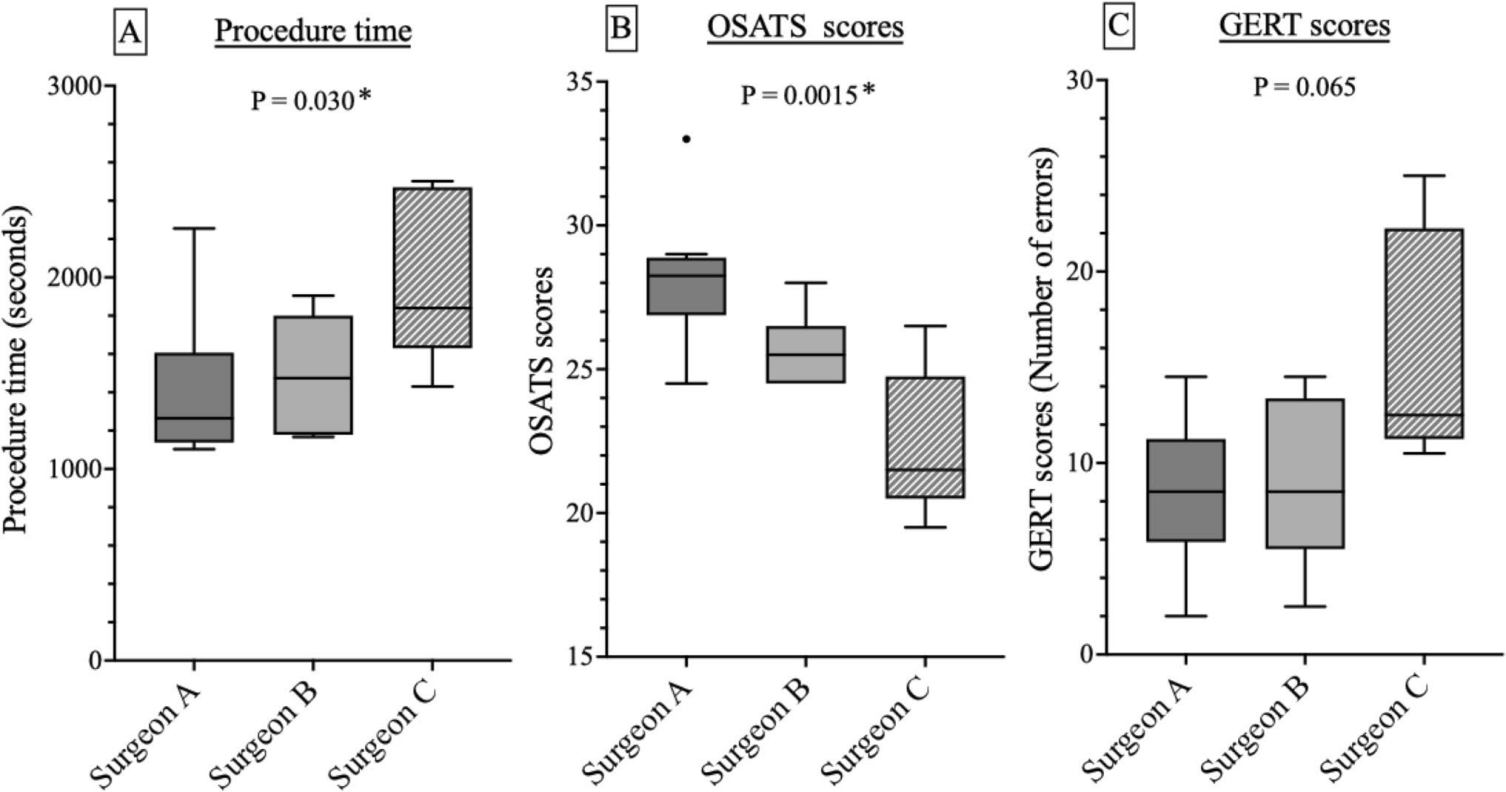
Subgroup analysis of surgeons performing ≥ 5 cases. **A** Procedure time. **B** Objective structured assessment of technical skill scores. **C** Generic error rating tool scores

In the subgroup analysis of surgeons who performed five or more cases (Surgeons A, B, and C), a significant difference was observed in procedure time (*P* = 0.030) and OSATS scores (*P* = 0.0015) as determined by the Kruskal–Wallis test. Although GERT scores also tended to vary among surgeons, the difference did not reach statistical significance (*P* = 0.065) (Fig. 4).

## Discussion

The study examined the technical performance of pediatric surgeons during SILPEC procedures using validated assessment tools—OSATS and GERT—and analyzed the relationship between technical performance scores, procedural errors/events, and surgical outcomes. This study presents three novel findings on the use of the GERT in pediatric laparoscopic surgery. First, GERT showed high inter-rater reliability in identifying intraoperative errors and events during pediatric SILPEC procedures. Second, a significant negative correlation was found between the number of errors identified by GERT and OSATS scores, indicating that GERT-derived metrics may serve as objective indicators of technical proficiency in SILPEC. Third, intraoperative errors and events were disproportionately concentrated in specific surgical steps, which may accurately reflect variations in the technical difficulty across different phases of the procedure.

Systematic recording of intraoperative technical errors is crucial for improving patient outcomes, as even seemingly minor events can increase postoperative morbidity and mortality [14]. Furthermore, ongoing monitoring and feedback regarding intraoperative errors have been shown to enhance technical performance and reduce adverse events [15]. In applying GERT to pediatric SILPEC procedures, this study achieved excellent inter-rater reliability, with ICCs exceeding 0.90 for both errors and events. This high level of consistency aligns with the findings of prior studies in adult laparoscopic surgery, wherein ICCs greater than 0.85 were reported [8, 9], and extends the use of GERT to pediatric laparoscopic surgery. However, certain tasks in our study, such as abdominal access/removal of instruments (ICC = 0.54) and suturing (ICC = 0.11), exhibited lower inter-rater reliability. Similar challenges were reported in a previous study, where lower frequencies of observed errors contributed to reduced agreement between raters [8]. Also, more explicit operational definitions are required to distinguish between actions reflecting the surgeon’s intended technique and those representing a deviation from the intended actions—i.e., an error—to avoid ambiguity. Likely, both the infrequency of errors and the inherent subjectivity in classifying certain actions contributed to the diminished inter-rater reliability observed in these task groups. A systematic understanding of the types of errors that may occur, as well as the time, reason, and frequency of their occurrence, is essential in effectively incorporating error-related information into surgical education [16]. In this study, the use of GERT enabled structured identification and documentation of such error characteristics even in pediatric laparoscopic surgery. These findings suggest that GERT may serve as a foundation for future educational frameworks, facilitating individualized feedback and promoting more effective technical skill development through the visualization of intraoperative errors.

The present study, consistent with prior studies [8, 9], demonstrated a significant negative correlation between the number of intraoperative errors and events identified by GERT and the OSATS global performance scores. This suggests that higher-rated technical performance ratings are associated with fewer procedural errors, supporting the validity of GERT-derived metrics as reliable indicators of surgical proficiency. Historically, surrogate metrics such as surgeon experience, surgeon volume, operative time, and intraoperative blood loss have been used [17–19]. However, these measures are confounded by patient- or context-specific factors and do not directly assess technical skill. In contrast, OSATS provides a widely accepted and validated framework for structured assessment of global technical performance, applicable in both live and video-based settings [20, 21]. However, despite its reliability, OSATS may lack the granularity needed to capture subtle intraoperative errors or procedural inefficiencies and therefore may offer limited support for providing targeted feedback [9]. GERT addresses this limitation by systematically identifying, categorizing, and quantifying specific technical errors and intraoperative events. Through such detailed error profiling, GERT offers a complementary and highly objective dimension to operative performance evaluation, capturing aspects of surgical execution that often elude global rating scales. These findings highlight the potential of GERT-derived error metrics to serve as valuable indicators of technical proficiency in pediatric SILPEC, particularly when used alongside established tools such as OSATS.

Our analysis revealed that intraoperative technical errors and events were not evenly distributed throughout the procedure but rather disproportionately concentrated in certain steps—most notably Step 3. This step-specific clustering pattern of errors is consistent with observations from laparoscopic procedures in previous reports. For example, Bonrath et al. found that novice surgeons made significantly more errors than experts during the most complex phases of a laparoscopic gastric bypass, indicating that error rates can spike at particularly challenging steps [8]. Likewise, in gynecologic laparoscopy, Husslein et al. identified the “vault closure” phase of a hysterectomy as the most error-prone step, attributing this high error rate to the requirement of advanced intracorporeal suturing skills [9]. In pediatric laparoscopy, Miyake et al. noted that peritoneal injuries and skips during LPEC frequently occur while passing the testicular vessels and spermatic duct [22]—a maneuver corresponding to Step 3 in our series—further underscoring the inherent technical difficulty of this phase of the operation. These examples illustrate how GERT-based analysis allows visualization of not only overall technical proficiency but also nuanced differences in performance at specific procedural steps, often mirroring the varying complexity of these steps. Recognizing these challenges allows surgeons to anticipate potential difficulties and allocate cognitive resources effectively, thereby enabling more precise error mitigation [23]. Furthermore, such granular insight—which may not be captured by traditional global rating scales—is invaluable for surgical education, as it enables targeted feedback and training focused on the most error-prone stages of the procedure, thereby addressing step-specific skill deficiencies and ultimately enhancing operative proficiency [6, 24].

This study has some limitations. First, the study was conducted at a single institution using a retrospective design, which might have introduced selection bias or unmeasured confounders, limiting the robustness of the findings. Further, while the sample size was determined to be adequate based on a priori power analysis, caution is warranted when extrapolating our results to other settings or larger populations. Second, although formal rater training for OSATS is recommended to strengthen validity evidence—particularly within the framework proposed by Messick—no additional OSATS-specific training was conducted for this study. However, both evaluators were board-certified pediatric surgeons with extensive experience in SILPEC and prior exposure to the OSATS framework in educational settings. Given the intuitive and widely adopted nature of the global rating scale, we considered their shared understanding and expertise sufficient for consistent assessment in this context. Finally, we intentionally focused on the SILPEC procedure, a relatively simple and task-homogeneous pediatric laparoscopic surgery, to ensure consistent and reliable assessment using GERT. However, it remains to be determined whether the performance patterns and error profiles observed in SILPEC can be generalized to more complex pediatric procedures involving greater task variability and technical challenges. Future studies should aim to validate the applicability of the GERT framework in diverse pediatric surgeries and explore advanced evaluation approaches, such as AI-driven video analytics, to enable objective and scalable surgical performance assessments.

In conclusion, this study demonstrated that GERT is an effective tool for assessing technical performance in pediatric laparoscopic surgery, specifically during SILPEC procedures. GERT’s error-based framework goes beyond scoring by allowing detailed identification and classification of intraoperative errors and events. This enables targeted feedback tailored to individual technical challenges. Such feedback can support deliberate practice and continuous skill improvement, ultimately advancing pediatric surgical education.

## Data Availability

The data used and/or analyzed during the current study are available from the corresponding author upon reasonable request.
